# Reduced Brain Complexity During the Execution of a Sequential Arm Movement Following an Augmented‐Feedback‐Based Practice: A Multiscale Entropy Analysis

**DOI:** 10.1111/ejn.70729

**Published:** 2026-10-22

**Authors:** Daghan Piskin, Linda Margraf, Matthias Weigelt, Jochen Baumeister

**Affiliations:** ^1^ Department Sports & Health, Exercise Science & Neuroscience Paderborn University Paderborn Germany; ^2^ Department Sports & Health, Psychology and Movement Science Paderborn University Paderborn Germany

**Keywords:** brain complexity, EEG, motor learning, multiscale entropy

## Abstract

Motor learning is accompanied by adaptive changes in neural processing, yet the extent to which feedback‐based practice alters the complexity of cortical activity during movement execution remains unclear. The present study examined learning‐associated changes in brain complexity during the execution of a sequential arm movement following augmented‐feedback‐based practice. Thirty‐eight healthy young adults practiced a sequential arm movement with augmented feedback across five sessions. Cortical activity was recorded with electroencephalography during the first and last practice sessions. Multiscale entropy was used to quantify brain complexity across 50 time scales. Behavioral measures indicated improved movement accuracy and reduced dual‐task costs across practice. At the cortical level, a significant global decrease in entropy was observed from the first to the last practice session. Further analyses revealed that the differences were confined to coarse temporal window and midline frontal electrodes Fz and FCz. In addition, changes in coarse‐scale entropy at FCz were negatively correlated with reductions in absolute error. Together, the present findings suggest that augmented‐feedback‐based practice is accompanied by a refinement of cortical complexity during movement execution, particularly in large‐scale frontal dynamics. Multiscale entropy may therefore provide a sensitive framework for capturing learning‐related neural adaptations that complement conventional electroencephalographic measures and advance the understanding of motor learning at the systems level.

## Graphical abstract

Augmented‐feedback‐based practice of a sequential arm movement was accompanied by improved behavioral performance and a global reduction in EEG multiscale entropy. This reduction was most evident at coarse temporal scales (31–50), with significant effects at the midline frontal channels Fz and FCz. Coarse‐scale entropy change at FCz was negatively correlated with change in absolute error.

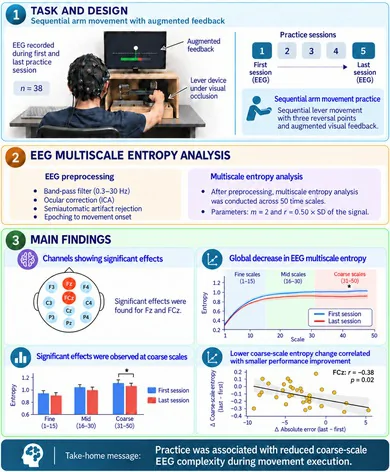

**Abbreviations**
ACC: anterior cingulate cortex
AE: absolute error
ANOVA: analysis of variance
EEG: electroencephalography
ERP: event‐related potential
MSE: multiscale entropy
SD: standard deviation
SEM: standard error of the mean
SMA: supplementary motor area

## 1 Introduction

Motor learning represents the set of internal processes associated with practice or experience that lead to relatively permanent changes in the capability for skilled movement (Seidler 2010). Beyond athletic performance, the capacity to internalize novel motor sequences is a fundamental pillar of human autonomy across a wide range of settings from the mastery of complex vocational skills to the restoration of functions in rehabilitation contexts (Krakauer 2006; Wong and Matsumoto 2008). As an individual transitions from explorative, cognitively demanding attempts to an automated, fluid motor execution, the associated neural mechanisms undergo continuous, adaptive reorganization (Dayan and Cohen 2011; Schmidt 2003; Stergiou and Decker 2011). This adaptation of neurophysiological mechanisms serves as the essential driver for performance improvements and long‐term retention that define successful learning. Consequently, characterizing these mechanisms that facilitate skill acquisition in motor behavior is essential for both optimizing specialized training protocols and developing targeted clinical interventions for motor recovery (Dayan and Cohen 2011; Krakauer 2006).

Augmented feedback has emerged as one of the most frequently employed training paradigms to promote motor learning. Although task‐intrinsic feedback arises inherently from the task execution itself providing information related with the sensory consequences of a movement, augmented feedback delivers extrinsic, performance‐related information from an external source, such as knowledge of results or knowledge of performance (Ranganathan and Newell 2010). This external information supplements task‐intrinsic sensory feedback and facilitates error‐detection (Schmidt and Lee 2020; Sigrist et al. 2013). Depending on the augmented feedback design, different mechanisms, such as supervised or reinforcement learning, yield neural adaptations that gradually enhance performance (Caligiore et al. 2019). By precisely informing the learner of the discrepancy between their current execution and the goal of the task, quantitative augmented feedback (e.g., magnitude and/or direction of errors) directly engages neural mechanisms responsible for comparing actual motor output with the intended motor plan (i.e., supervised learning). Beyond its informational content, the valence of augmented feedback, signaling whether success (positive) or failure (negative), significantly contributes to motor learning by modulating motivational and error‐processing pathways (i.e., reinforcement learning) (Krause et al. 2020; Krigolson et al. 2015; Zobe et al. 2019). Although positive feedback supports the reinforcement of successful movement patterns and improves self‐efficacy, negative feedback provides a critical signal for behavioral adjustment, triggering corrective mechanisms and attentional processes to minimize future performance discrepancies (Krause et al. 2020; Margraf et al. 2022a; Wulf and Lewthwaite 2016).

The progression of motor learning through these evaluative processes is reflected in distinct neurophysiological signatures that characterize the shift from effortful, cognitive monitoring toward a more fluid, automatized execution of a movement sequence (Hikosaka et al. 1999; Margraf et al. 2022a, 2022b, 2023). As research on learning‐associated changes in the motor domain has grown, traditional electroencephalographic (EEG) metrics, such as event‐related potentials (ERP) and oscillatory power, have revealed adaptations in both feedback‐related evaluative (Colino et al. 2020; Faßbender et al. 2023; Krause et al. 2020; Margraf et al. 2022a, 2022b, 2023) and execution‐related motor processes (Beaulieu et al. 2014; Lum et al. 2023; Pollok et al. 2014). These adaptations are commonly interpreted as reflecting a reduced reliance on attention‐demanding performance monitoring and cognitive control as movement execution becomes increasingly automated and predictable (McDougle et al. 2016; Stergiou and Decker 2011). Although traditional magnitude–based measures, such as averaged ERPs and spectral power, are highly informative for specific learning‐related mechanisms, they do not fully capture the rich temporal organization of the underlying neural signal (Heisz and McIntosh 2013; Hutka et al. 2013). By quantifying the complexity of the temporal structure as an information processing proxy of the brain, nonlinear EEG measures can provide a complementary account of how neural information processing is refined during the acquisition of skilled movement (Lau et al. 2022; Piskin, Müller, et al. 2026; Wang et al. 2018).

Entropy analysis presents a sophisticated framework for quantifying the regularity and predictability of EEG time series, which enables an operational measure of their temporal structure complexity (Keshmiri 2020). Although traditional entropy measures are often limited to a single time scale, multiscale entropy (MSE) captures the complexity of biological systems more comprehensively by quantifying signal regularity and predictability across multiple temporal scales using a coarse‐graining procedure (Costa et al. 2002, 2005). Increasing the scale factor progressively attenuates faster fluctuations and places relatively greater emphasis on slower temporal structure expressed over longer intervals. Therefore, in the context of MSE, fine scales are interpreted as local information processing, whereas coarser scales are interpreted as the integration of information into long‐range networks (McIntosh et al. 2014; Vakorin et al. 2011). The resulting scale profile can therefore reveal effects confined to particular temporal resolutions that could be obscured by a single entropy estimate. Capturing a complexity profile that extends across multiple time scales may reveal how local and distributed information processing is modulated by automatized movement execution and serve as a sensitive indicator of the system's functional state and capacity to adapt (McIntosh et al. 2014; Mizuno et al. 2010; Vakorin et al. 2011; Wang et al. 2020).

Preliminary evidence from the emerging field of movement‐related MSE suggests that brain complexity is a dynamic feature that is potentially influenced by expertise, practice, and performance outcome with effects frequently differing across temporal scales and cortical regions. For instance, expert football players exhibited significantly reduced cortical entropy compared with novices while kicking, with lower entropy levels being negatively associated with accuracy performance and showing spatial and scale‐specificity (Piskin, Cobani, et al. 2026). Similarly, across 4 weeks of exergame practice, improvements in older adults' gameplay were accompanied by distributed reductions in task‐related entropy that were concentrated at coarse scales, suggesting a practice‐related refinement of information integration into distributed networks (Piskin, Müller, et al. 2026). Although these preliminary findings show a trend toward reduced brain complexity during task execution with enhanced motor performance, this relationship may differ during movement preparation, as successful golf putts were preceded by higher entropy compared with unsuccessful putts in the preparatory phase at selected scales over parietal and occipital sites in the study of Chueh et al. (2025). Therefore, the existing findings do not indicate a universally optimal direction of complexity associated with motor performance, but rather scale‐specific increases or decreases depending on the task phase, regional dynamics, and degree of skill acquisition. This context dependence is further supported by findings in football players following anterior cruciate ligament reconstruction, who demonstrated reduced kick‐related cortical entropy at medium and coarse scales alongside changes in repeated kicking behavior compared with their healthy counterparts, suggesting a modulation in slower temporal dynamics as a result of adaptive behavior (Piskin et al. 2025).

Despite these initial insights, it remains unclear if augmented‐feedback‐based practice of a sequential arm movement can also modulate task‐related brain complexity besides previously described changes in linear dynamics associated with feedback evaluation (Margraf et al. 2022a, 2022b, 2023). Exploring how feedback‐driven skill acquisition alters the temporal structure of the underlying neural signal using MSE can help understand how the adjustment and reinforcement of movement patterns dynamically reorganize the local and long‐range information processing in the brain and contribute to existing evidence regarding motor performance and brain complexity. Therefore, the current study aims to explore learning‐associated changes in brain complexity during the execution of a sequential arm movement following augmented‐feedback‐based practice. In line with previous research, we hypothesize to find reduced entropy values particularly at mid‐to‐coarse scales (Cnudde et al. 2021; Piskin, Cobani, et al. 2026; Piskin, Müller, et al. 2026).

## 2 Methods

The current study is an ex‐post‐facto (re‐) analysis of a neural dataset as part of a study investigating valence‐dependent neural correlates in a sequential arm movement task with augmented feedback that has already been published (Margraf et al. 2022a, 2022b, 2023). Therefore, essential aspects of the experimental setup and the task, previously detailed in the cited works, are summarized here in a focused manner. Aspects of the new analysis are described in detail. The present study was preregistered with the number 258772 (https://aspredicted.org/tn5wa5.pdf).

### 2.1 Participants

The final sample size consisted of 38 undergraduate students totally (20 females; mean age = 20.89 ± 1.68; predominantly right‐handed, no orthopedic limitations, normal or corrected to normal vision). All participants gave informed consent prior to participation. Ethical approval was obtained for all procedures. For more details, see Margraf et al. (2022a).

### 2.2 Apparatus and Task

Participants practiced a sequential arm movement executed with the right arm using an adjustable forearm lever device (Figure 1). The required movement sequence involved three defined reversal points (70°, 20°, and 70°), which had to be executed within a maximum movement time of 1800 ms. Visual movement control was intentionally restricted. More details about the criterion motor task and the experimental setup can be found in Margraf et al. (2022a).

**FIGURE 1:**
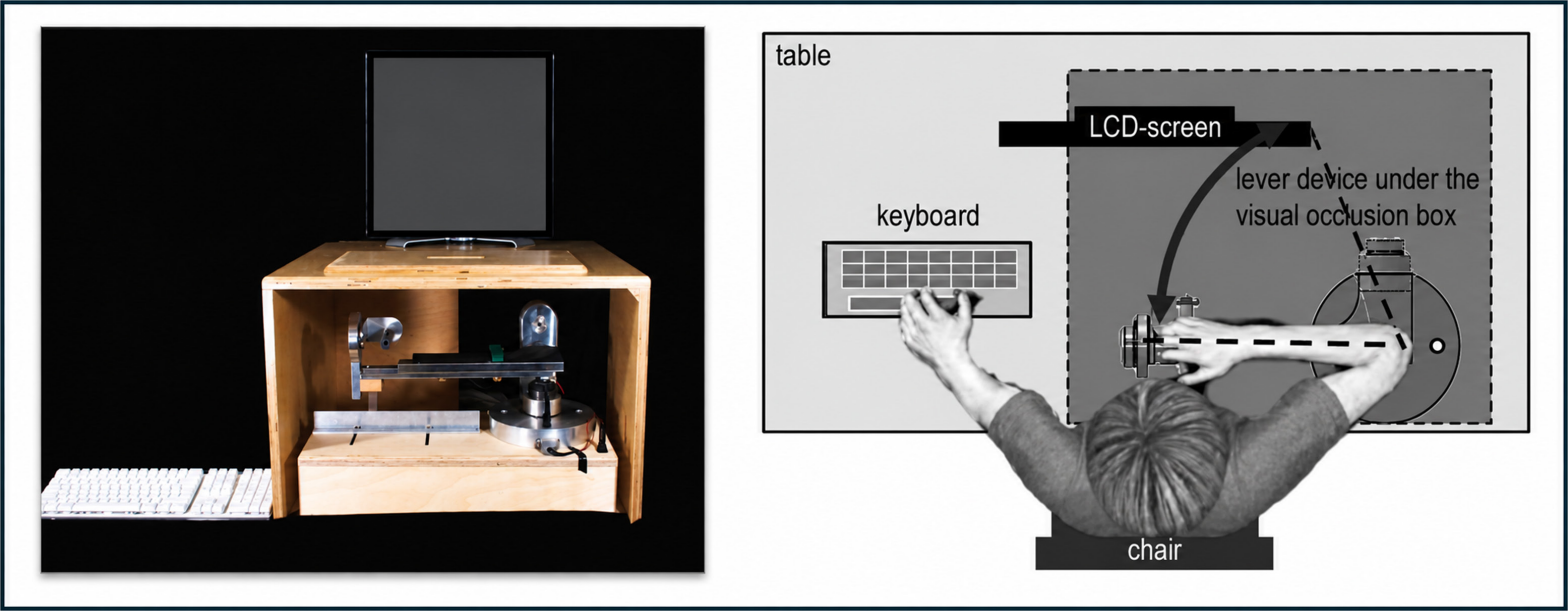
Experimental setting. On the left side, the setup is shown with the lever‐device placed under the wooden box, which serves also as a podium for the monitor. On the right side, a participant is sitting in front of the setup with the right arm placed on the lever in the starting position and with the left hand on the keyboard as an input device to start the task. From the starting position, participants move the lever to 70°, from 70° to 20°, back to 70°, and finish the movement by returning to the starting position (reprinted from Margraf et al. 2022a, copyright [2021], with permission from Elsevier).

### 2.3 Procedures

The participants completed seven experimental sessions within 14 days with at least 24 h between successive sessions. The extensive practice intervention comprised five sessions with 192 practice trials each. Motor learning and the degree of motor automatization were assessed three times (in Session 1 as a pre‐acquisition test, in Session 2 before the first practice as a pre‐test, and in Session 7 as a retention test 24 h after the last practice) using a dual‐task paradigm in which a visuospatial two‐back task served as an additional cognitive task. However, as the current analysis focuses solely on the practice intervention, no further information will be provided here. For more details about the dual‐task tests, see Margraf et al. (2022b).

During the five practice sessions, participants received augmented feedback after every trial. The feedback was displayed as a bar graph indicating the direction and the magnitude of the error for the reversal with the largest deviation from the goal value. There was no information about which reversal was concerned (the first, the second, or the third one). The feedback was based on the performance adaptive bandwidth for positive feedback. The criterion for positive feedback was dynamically set according to the median performance in preceding trials, resulting in an (almost) equal frequency of positive and negative feedback trials. Optically, the bandwidth was displayed as a transparent green bar that overlaid the blue error bar. One hundred points were awarded for hitting the bandwidth, whereas 100 points were deducted for missing it. EEG was recorded during the first and last practice sessions. More details about the structure of the practice sessions and the rationale of the feedback design can be found in Margraf et al. (2022a).

### 2.4 Behavioral Data Analysis

All trials that contained less or more than three reversals were excluded from the analysis. The absolute error (AE) was defined as measurement of movement accuracy calculated as the difference between the actual and goal values. This difference was calculated for each of the three reversals; the mean of the differences was then used as the AE of this trial. The degree of motor automatization was evaluated based on the dual‐task tests with the pretest directly before the first practice and the retention test at least 24 h after the last practice. To measure the degree of motor automatization, dual‐task costs were calculated for the cognitive task defined as the difference between errors that were made in the single‐task and dual‐task conditions. For a more detailed description, see Margraf et al. (2022a, 2022b).

### 2.5 EEG Recording

Cortical activity was recorded using a 16‐channel EEG system (V‐amp, Brain Products, Munich, Germany) applied with Ag/AgCl electrodes according to the 10–20 system (actiCAP, Brain Products). Active electrodes were placed on F3, FCz, Fz, F4, Cz3, Cz, C4, P3, Pz, and P4 with the ground electrode on FPz and online reference on FC6. Additional electrodes were placed on each mastoid, laterally of both eyes as well as above and below the right eye. Impedances of all electrodes were kept below 20 Ω before starting data acquisition. The data were recorded continuously at a sampling rate of 500 Hz (Brain Vision Recorder 2.0, Brain Products, Munich, Germany). A trigger was sent with the onset of augmented feedback presentation via a photodiode (BPW21R, Vishay) that was attached to the right lower corner of the feedback screen.

### 2.6 EEG Preprocessing and MSE Analysis

Preprocessing of the EEG data was conducted with Brain Vision Analyzer 2.0 (Brain Products, Munich, Germany). The data were re‐referenced to average mastoids and offline filtered with a 0.3–30‐Hz zero phase shift Butterworth filter. Line noise of 50 Hz was also removed via notch filter. The semiautomatic mode of the ocular correction algorithm of the analyzer was used to correct ocular artifacts based on independent component analysis. Artifact rejection was done with the semiautomatic mode of the artifact rejection algorithm of the analyzer (maximal allowed voltage steps: 50 μV/ms; maximal allowed difference of values in interval: 200 μV; lowest allowed activity: 0.5 μV). The triggers set by the photodiode were manually adapted to the movement onset and imported as events considering the time window of the movement. The events were reconstructed based on the experimental setup (Margraf et al. 2022a, 2022b, 2023). In the experimental software (Padutas), a movement window of 3 s was defined. The end of this movement window was the timepoint of feedback onset triggered by the photodiode. The software also reports the timepoint of leaving the 0° position (movement onset) in relation to the start of the Dasylab measurement. Based on this information, the movement onset triggers were calculated. EEG data were then epoched time‐locked to movement onset with regard to this time window, resulting in an epoch length of 7 s (−4 to 3 s). The length of the epochs (especially the long pre‐trigger interval) was chosen to have a visual marker that the triggers were correctly set. In the original study, it was not planned to analyze the movement window, so there were no markers to identify movement onset and offset. Entropy analysis was performed on the movement execution segment (0 to 3 s) using MATLAB (R2025a, The MathWorks, United States).

To quantify brain signal complexity, a modified MSE analysis was applied to the preprocessed EEG data using custom‐built scripts. The methodology established by Grandy et al. (2016) for sparse EEG data was employed. The execution phase (0–3 s) of the epochs was considered for analysis. For computational efficiency, 20 epochs were randomly selected for each dataset and concatenated with a total of 30,000 sample points (20 epochs × 3 s × 500 sample points). This data length complies with the previously reported requirements for reliable entropy estimates (Grandy et al. 2016; Gow et al. 2015; Piskin, Cobani, et al. 2026). From these time series, 50 distinct time scales were generated using a within‐segment coarse‐graining procedure, defined as:

yjT=1T+∑i=j−1T+1jTxi,1≤yj≤NT

where *Τ* represents the time scale, *y*_*j*_ denotes the constructed time series, *x*_*i*_ is a data point in the original signal, and *N* is the total length of the time series (Costa et al. 2002). Entropy was then estimated for each channel and scale on the residuals of the signal after subtracting the within‐average and accounting for patterns that did not exceed segment borders (Grandy et al. 2016; Puglia et al. 2022). The following formula was used:

$$S_{E}\left(m,N,r\right)=\ln\frac{A}{B}=\ln\frac{\sum_{i=1}^{N-m}n_{i}^{m}}{\sum_{i=1}^{N-m}n_{i}^{m+1}}$$

where *m* is the embedding dimension, *r* is the similarity criterion, and *A* and *B* represent the probability that two sequences of length *m* and *m* + 1 remain similar within the threshold *r* (Costa et al. 2002). Following established protocols, the embedding dimension was set to *m* = 2 (Costa et al. 2002, 2005; Grandy et al. 2016). To prevent the entropy estimates from being biased by changes in signal variance across coarser scales, the similarity criterion was dynamically adjusted for each scale as *r* = 0.50 × SD of the coarse‐grained time series (Kosciessa et al. 2020; Puglia et al. 2022). This resulted in a 50 × 10 entropy matrix (scales × channels) for each participant and practice session.

### 2.7 Statistical Approaches

In the original study, the behavioral results were analyzed based on ANOVA with repeated measures on time (pre‐acquisition test, pretest, retention test) and condition (single‐task, dual‐task), separately for the AE in the motor task as measurement of accuracy and for the n‐back error to estimate the amount of automatization with an alpha level of 0.05 and partial eta squared as effect size. Follow‐up analyses were conducted with paired *t*‐tests.

Statistical inference for the EEG entropy data was performed using paired sign‐flip permutation tests. The permutation framework is a robust nonparametric approach for EEG data as inference is based on the empirical permutation distribution under the null, rather than on Gaussian assumptions, making it suitable for high‐dimensional and potentially non‐normally distributed electrophysiological measures (Nichols and Holmes 2002; Maris and Oostenveld 2007). For every participant, entropy differences were calculated as entropy_*last session*_ − entropy_*first session*_, with negative values indicating lower entropy following practice. For a detailed channel and scale‐wise analysis, a paired *t*‐statistic was calculated from the participant‐level differences at each of the 50 scales and 10 channels. Under the null hypothesis, permutation distributions were generated by randomly reversing the sign of each participant's paired difference. The same participant‐level sign vector was applied simultaneously across all 500 channel–scale combinations in each permutation to retain the dependence structure of the data. Family‐wise error was controlled across all 500 channel–scale comparisons using a single‐step permutation max‐*T* procedure. In each permutation, the maximum directionally oriented *t*‐statistic across all channel–scale combinations was retained. Because the differences were calculated as entropy_*last session*_ − entropy_*first session*_, this procedure is computationally equivalent to retaining the minimum raw *t*‐value in each permutation.

To provide a lower dimensional summary of changes extending across adjacent scales and enhance interpretability, an additional post‐registration analysis (Nosek et al. 2018) grouped 50 scales into three time windows. Scales 1–15, 16–30, and coarse 31–50 were defined as fine, mid, and coarse time windows, respectively. Similar aggregation of EEG MSE estimates into broad scale ranges has been used previously to summarize scale‐dependent effects with no established guidelines for determining scale range bins (Angulo‐Ruiz et al. 2023; Gu et al. 2022). For each participant and channel, entropy was first averaged across the scales within each range. An initial omnibus test then evaluated the overall session difference after averaging across the 10 EEG channels and three scale ranges to address the a priori hypothesis of an overall entropy decrease. Due to single comparisons, no multiplicity corrections were performed. Subsequently, separate window‐level tests were conducted after averaging entropy across the 10 EEG channels within each scale range. Multiple comparisons across the three window‐level tests were controlled using the same directionally oriented permutation max‐*T* procedure. Effect sizes for the omnibus, scale‐wise, and window‐level comparisons were expressed as Cohen's *dz*.

To characterize the sensor‐level distribution of effects identified by the window analysis, a follow‐up analysis was conducted within each window that survived the window‐level correction. Entropy was averaged across the relevant scales separately for each EEG channel, and a directional paired sign‐flip permutation test was performed at each of the 10 channels. The resulting permutation‐derived *p*‐values were adjusted across channels using the Benjamini–Hochberg false‐discovery‐rate procedure (Benjamini and Hochberg 1995). Because this conditional channel‐wise analysis intended to characterize the sensor‐level distribution of an effect already identified within the time window, the Benjamini–Hochberg procedure was selected to control the expected proportion of false discoveries among rejected channel‐level hypotheses and retain greater sensitivity than an additional family‐wise‐error correction (Groppe et al. 2011). All permutation analyses were based on 10,000 random sign flips. Statistical significance was defined as *p* < 0.05.

As an exploratory secondary analysis, associations between learning‐related changes in movement accuracy and entropy were examined for channels showing significant effects in the follow‐up analysis. Entropy change was calculated as entropy_*last session*_ − entropy_*first session*_ after averaging entropy estimates across scales in the time window. Behavioral change was analogously calculated as the later measurement minus the earlier measurement such that negative absolute‐error values indicated improved accuracy. After verifying that data distributions did not show deviations from normality (Lilliefors 1967), Pearson correlations were calculated separately for channels with significant effects. Given the one‐dimensional data, the correlation tests were based on the conventional parametric sampling distribution.

## 3 Results

### 3.1 Behavioral Findings

For a detailed description of the behavioral results, refer to Margraf et al. (2022b). In the following, a brief summary of the main results with respect to this reanalysis is presented. Regarding AE in the motor task, the results revealed that participants significantly enhanced accuracy already from the pre‐acquisition test to the pretest and to the retention test. There were no further improvements from the pretest to the retention test. Although participants hit the reversals more precisely in the single‐task condition compared with the dual‐task condition, analysis of the dual‐task costs focused on the cognitive task as there were differences in motor dual‐task costs in the three tests. With respect to the cognitive task, the results revealed that participants significantly improved their performance from the pre‐acquisition test to the pretest and from the pretest to the retention test. Further, participants performed better in the single‐task condition as compared with the dual‐task condition in all three tests. The dual‐task costs were significantly reduced in the retention test as compared with the pretest.

### 3.2 EEG Findings

Channel‐ and scale‐wise analyses demonstrated a decreasing trend in entropy from the first to the last practice sessions. Only Fz survived multiple‐comparison correction at scales 37, 40, and 45. Detailed findings are presented in the Supporting Information.

Omnibus analyses on the EEG data confirmed the significant global decrease in entropy (mean difference = −0.0212, *t* = −1.8074, *d*z = −0.2932, *p* = 0.0377). At the window level, mean differences were −0.0151 for scales 1–15, −0.0201 for scales 16–30, and −0.0283 for scales 31–50. After correction across windows, only the coarse window (scales 31–50) showed a significant negative effect (*t* = −2.1330, corrected *p* = 0.0347, *d*z = −0.3460), whereas the lower and intermediate windows did not survive correction (corrected *p*s ≥ 0.05) (Figure 2). The scalp map of averaged entropy estimates for each condition, scale window, and their differences is presented in Figure 3.

**FIGURE 2:**
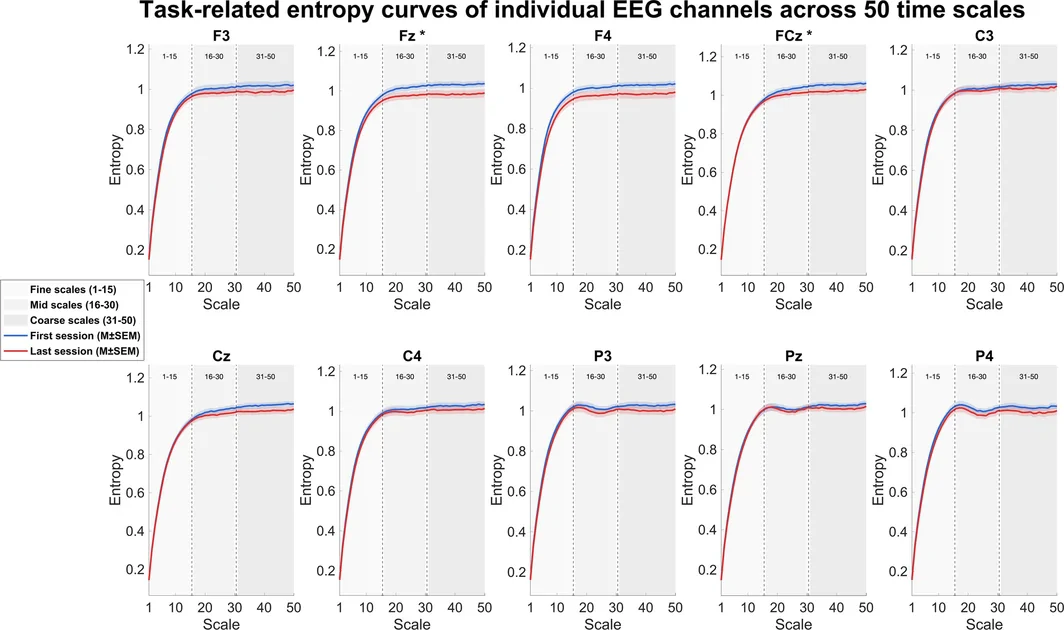
Entropy curves across EEG channels. Shaded areas indicate analyzed windows (1–15: fine scales, 16–30: mid scales, 31–50: coarse scales). The asterisks represent the channels showing significant decrease in entropy estimates in the secondary coarse‐window analysis. SEM, standard error of mean.

**FIGURE 3:**
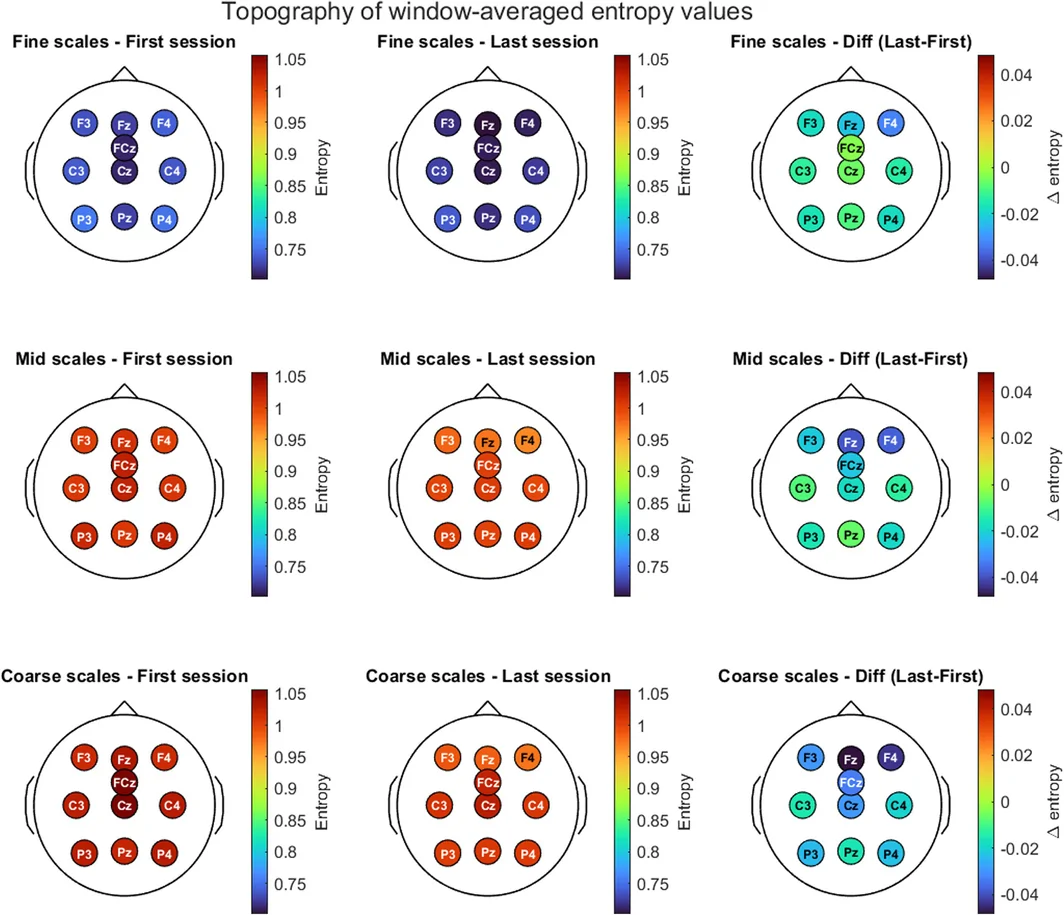
Scalp topography of entropy estimates for the first (left column) and the last practice session (mid column) for all three analyzed scale windows. Lighter colors in the mid column indicate reduced entropy. The right column shows the topography of observed differences between the sessions with largest values at coarse scales.

In the channel‐wise follow‐up analysis within the coarse window, significant negative effects after correction were observed at Fz (mean difference = −0.0484, *t* = −2.8903, corrected *p* = 0.0280, *d*z = −0.4689) and FCz (mean difference = −0.0334, *t* = −2.6059, corrected *p* = 0.0280, *d*z = −0.4227) (Figure 4).

**FIGURE 4:**
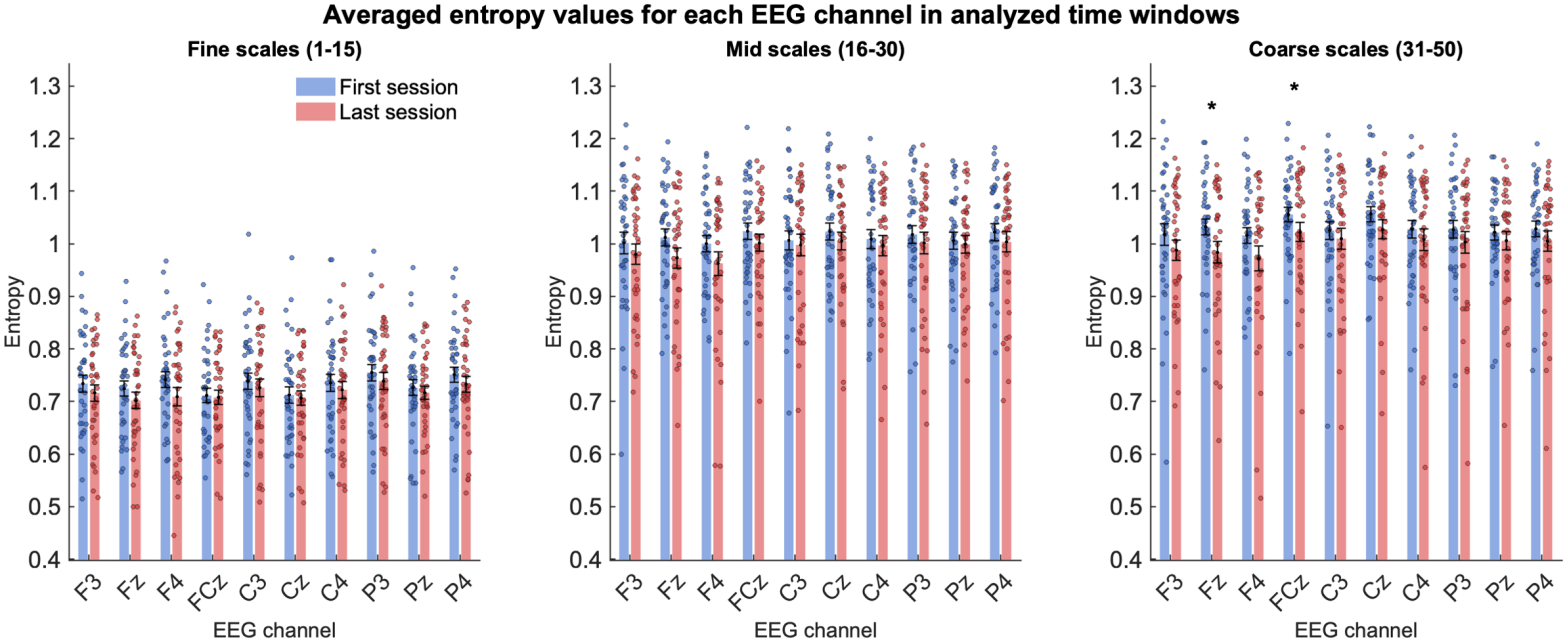
Window‐wise averaged entropy values for each individual channel. Despite the global trend toward lower entropy, only Fz and FCz channels demonstrated significant decreases from the first to the last practice sessions.

The secondary analysis revealed a significant moderate negative correlation between AE in dual‐task condition and coarse entropy of FCz (*r*(36) = −0.38, *p* = 0.02). Higher reduction in errors was correlated with a smaller reduction in entropy. Fz did not show any significant correlation (*r*(36) = −0.29, *p* = 0.08). Scatterplots are presented for both channels in Figure 5.

**FIGURE 5:**
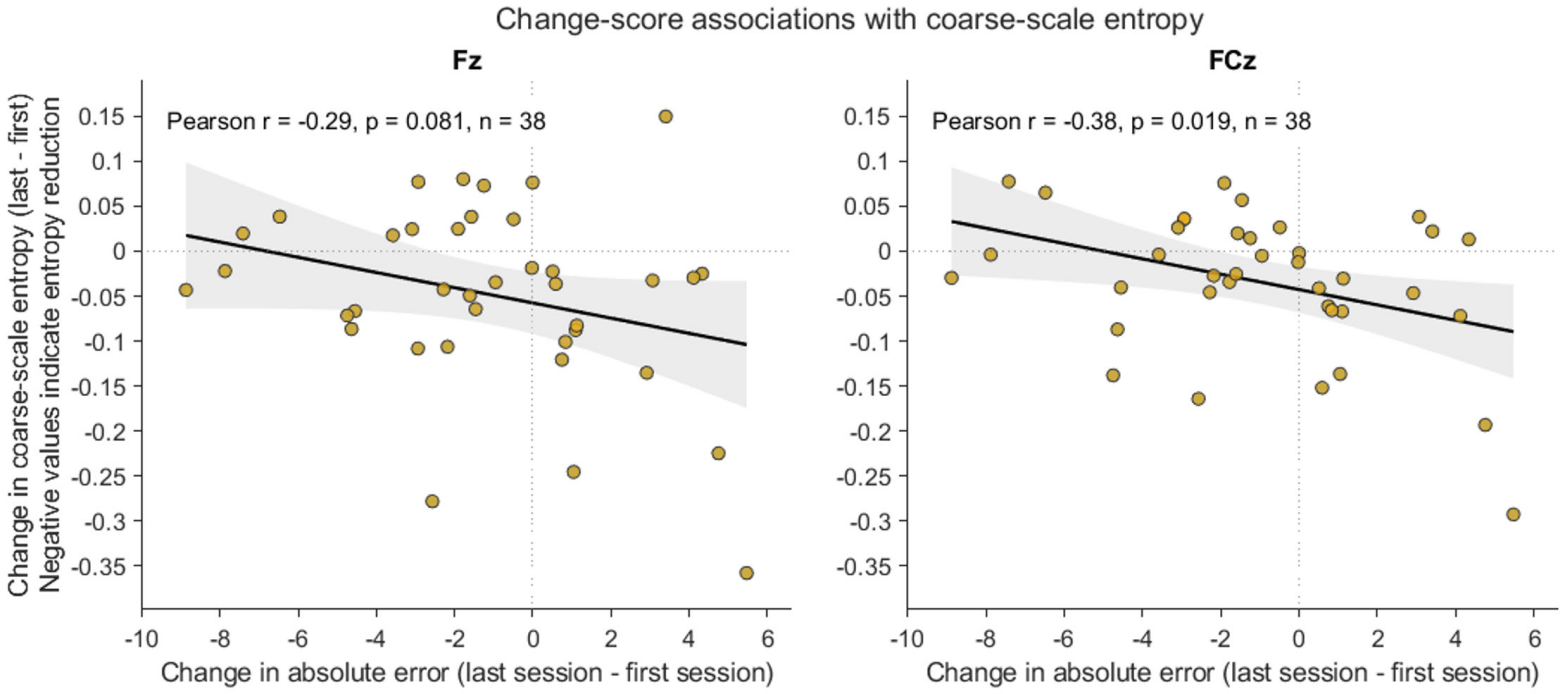
Associations between learning‐related changes in coarse‐scale entropy and absolute error during the dual‐task condition. Scatterplots show individual participant change scores for entropy at Fz and FCz averaged across scales 31–50, plotted against changes in absolute error. Entropy and absolute error changes were calculated as last practice session—first practice session with negative values indicating reductions.

## 4 Discussion

The present study examined learning‐associated changes in brain complexity during the execution of a sequential arm movement following augmented‐feedback‐based practice. As reported in the previous study with the same dataset (Margraf et al. 2022b), practice of five sessions yielded improvements in behavioral measures. At the cortical level, enhanced performance was accompanied by a significant reduction in entropy, localized over the midline frontal scalp regions and the coarse window. In addition, further analyses revealed a negative correlation between changes in AE and coarse window entropy with relatively smaller reduction in entropy coupling with a higher enhancement in accuracy.

### 4.1 Behavioral Findings

Behaviorally, the findings support that feedback‐based practice resulted in learning the executed sequence. As discussed in the previous report (Margraf et al. 2022b), participants improved relevant performance measures across the sessions, with gains in movement accuracy and consistency, and a reduction in dual‐task costs indicating a progression toward a more stable and automated execution of the practiced motor sequence. These behavioral adaptations are in line with established accounts of motor learning, according to which repeated practice gradually reduces the reliance on capacity‐limited attentional control and promotes more efficient and automatic movement regulation (Adams 1971; Fitts and Posner 1967; Haith and Krakauer 2018; Poldrack et al. 2005; Tsay et al. 2024). In the context of the present reanalysis, these improvements provide the functional backdrop against which the observed changes in brain complexity should be interpreted.

### 4.2 Entropy Changes From the First to the Last Practice Sessions

At the EEG level, the global omnibus analysis indicated an overall downregulation in brain complexity from the first to the last practice sessions. Although only Fz survived correction at channel‐ and scale‐wise analyses, window‐level and follow‐up analyses revealed that averaging across the correlated scales yielded a significant coarse‐window decrease in Fz and FCz, suggesting an aggregate effect in this time window. The observation that practice‐induced decreases in MSE were more evident in the midline frontal electrodes may be anatomically informative, as these two electrodes are commonly considered to measure scalp locations sensitive to frontal regions, such as the anterior cingulate cortex (ACC) and the supplementary motor area (SMA) (Gevins et al. 1997; Ogahara et al. 2023; Onton et al. 2005). The involvement of medial frontal regions including ACC and SMA during performance monitoring, voluntary movement preparation, and the internalization of action feedback has been documented consistently across neuroimaging studies (Cunnington et al. 2002; Li et al. 2018; Passingham et al. 2010; Ullsperger et al. 2014). Medial frontal regions have been reported to mediate cognitive‐motor integration detecting the discrepancies between predicted and actual task outcomes and scaling attentional allocation in proportion to the degree of behavioral uncertainty (Botvinick et al. 2004; Ridderinkhof et al. 2004). Moreover, frontal regions have also been shown to contribute to the temporal structuring of action sequences, the transformation of cognitive intentions into motor programs, and the gradual shift from externally guided to internally generated movement (Nachev et al. 2008; Ohbayashi 2021; Shima and Tanji 2000). Early motor learning relies on the intensive communication between frontal and sensorimotor regions, which together support the executive supervision of action selection, monitoring, and movement planning (Lohse et al. 2014; Rushworth et al. 2011). Consistent with learning‐associated consolidation processes described in neuroimaging studies, the effects observed in frontal entropy may reflect a decreased reliance on this supervision as movement execution becomes more efficient and increasingly automatic (Doyon et al. 2003; Hikosaka et al. 2002). The reduced reliance on supervision may manifest as changes in the temporal structure of frontal neural signals that may be detectable in these electrodes and align with previous findings indicating reduced frontal entropy with enhanced performance and learning (Cnudde et al. 2021; Piskin, Cobani, et al. 2026; Piskin, Müller, et al. 2026).

The scale‐specificity of the entropy decrease, which was most evident in the coarse window, helps characterize the temporal resolution at which the practice‐related effects may emerge. In the framework of MSE, coarse‐graining progressively attenuates fast local fluctuations and renders the entropy estimates incrementally sensitive to slower, more integrated signal components. Therefore, in the MSE analysis of EEG signals, coarser scales are interpreted as reflecting long‐range information integration across distributed networks at the macroscopic level rather than local, high‐frequency processing (Costa et al. 2005; Kosciessa et al. 2020; McIntosh et al. 2014; Vakorin et al. 2011). Extending the spatial distribution of the changes discussed previously, the stronger reduction in coarse‐window can be interpreted as a refined integration of information into distributed neural assemblies. During the initial phase of motor learning, medial frontal regions may likely interact more strongly with the associative circuits to supervise motor execution under enhanced monitoring (Hardwick et al. 2013). Such broader network‐level coordination may be expressed as greater irregularity of the signal at coarse temporal scales, as long‐range communication yields dynamically reconfiguring patterns of interregional synchrony, which consequently contributes to higher irregularity of the underlying signal (Kosciessa et al. 2020; Deco and Kringelbach 2016). During information exchange, distributed neural populations repeatedly couple and decouple, and the resulting alternation between phases of integration and segregation may enrich the temporal structure of slow dynamics (Tognoli and Kelso 2014), thereby contributing to elevated coarse‐scale entropy in the initial phase (Vakorin et al. 2011; Wang et al. 2018). However, as sequential performance becomes more automatic with practice, reliance on this distributed supervisory network and the information exchange across long‐range neural assemblies may decrease, potentially reflected as a more regular signal and lower coarse‐scale complexity (Ando et al. 2022; Doyon et al. 2003; Hikosaka et al. 2002; Vakorin et al. 2011; Wang et al. 2018). Previous findings have similarly reported practice‐associated changes particularly at coarse‐scale entropy (Cnudde et al. 2021; Piskin, Cobani, et al. 2026; Piskin, Müller, et al. 2026).

### 4.3 Correlation Between Changes in Entropy and AE

The correlation analysis may add nuance to the present findings, as it reveals that the association between performance improvement and complexity changes may not be strictly linear. Although coarse‐scale entropy decreased from the first to the last practice session, participants who showed a larger reduction in AE tended to exhibit a relatively smaller reduction in entropy. In this sense, the present findings suggest that successful motor learning may not be characterized by the strongest possible downregulation in brain complexity but rather by a balanced reorganization in which large‐scale dynamics are refined without being excessively reduced. This interpretation is compatible with theoretical accounts proposing that adaptive behavior depends on an equilibrium between neural stability and flexibility rather than on a simple shift toward maximal regularity (Cocchi et al. 2017; McDonough and Nashiro 2014; O'Byrne and Jerbi 2022). Although a certain degree of neural refinement is required to dismantle redundant cognitive processes and achieve automaticity, an excessive drop in entropy may expose neural control to an over‐stabilization or system rigidity. In the present context, it is conceivable that participants with stronger performance gains retained a functional degree of operational flexibility as the practiced sequence, despite repeated practice, likely required some level of monitoring, integration of prior feedback, and flexible adjustment. The present correlation does not necessarily contradict the overall reduction in entropy after practice, but rather suggests that the degree of reduction may vary across individuals depending on how efficiently neural control processes are reorganized during learning (Piskin, Cobani, et al. 2026). Given the effect observed only in one of the two analyzed channels and limited literature on learning‐associated entropy changes during movement execution, this finding should still be interpreted with caution.

Taken together, the present findings indicate that learning of a sequential arm movement through augmented‐feedback‐based practice is accompanied not only by behavioral improvements and neural changes associated with feedback processing as shown by our previous findings (Margraf et al. 2022a, 2022b, 2023) but also by a reduction of complexity in brain activity during task execution. More specifically, our data suggest that motor learning may be associated with a reduced complexity of midline frontal activity at coarse temporal scales, pointing to a more efficient and refined large‐scale control of execution as the practiced sequence became increasingly automatic. These findings contribute to the current literature by showing that MSE may capture learning‐related neural adaptations that reflect the reorganized integration of information across long‐range networks. At the same time, the present study contributes to a more differentiated understanding of how brain complexity alters with motor learning during movement execution, suggesting that practice‐related refinement may be expressed as a selective reduction in region‐ and scale‐specific dynamics. From a practical perspective, our findings provide preliminary evidence that brain complexity metrics may potentially serve as a sensitive indicator of learning‐related neural changes, a direction worth exploring in prospective studies about skill training, exercise interventions, and neurorehabilitation, where understanding the characteristics of neural adaptation is of particular relevance. Future work should clarify how these complexity changes evolve across different stages of learning and under different task and feedback demands.

### 4.4 Methodological Considerations and Prospects

Regarding the task and overall design, the present findings were obtained within a relatively short practice window despite the high number of trials, and with EEG recorded only at the first and last session within 14 days. Accordingly, the present data do not yet allow conclusions about the longer term trajectory of brain complexity across acquisition, consolidation, and retention. Learning‐associated complexity changes during the execution of a task may fluctuate across different stages of learning over a longer period of time, rather than demonstrating monotonical trends (Piskin, Cobani, et al. 2026). Future longitudinal studies should therefore track complexity at multiple time points and include delayed retention assessments to determine whether the present pattern reflects an early learning‐related refinement, a transient adaptation, or a more stable neural signature of skilled performance. In addition, the complexity correlates of enhanced performance may be task‐specific. Preliminary evidence suggests that different forms of motor learning may impact distinct cortical regions depending on the demands of the task (Piskin, Cobani, et al. 2026; Piskin, Müller, et al. 2026). Future work should therefore test whether the present frontal coarse‐scale pattern generalizes across motor tasks or whether other behavioral contexts are characterized by different spatial and temporal complexity profiles.

With regard to EEG‐specific aspects, the temporal interpretation of MSE scales depends on the retained sampling rate. Because scale factors are defined in samples, the same numerical scale may represent different intervals across studies with different sampling rates. Cross‐study comparisons should therefore consider the temporal interval represented by each scale (Courtiol et al. 2016). Complementary frequency–specific analysis or interpretations should also consider the Nyquist–Shannon sampling theorem (Shannon 1949), according to which the highest theoretically representable frequency is half the relevant sampling rate besides the bandwidth retained through filtering. Furthermore, there are no established guidelines for determining the bins of scale ranges, which leaves the description of time windows such as fine and intermediate vague except the physiological interpretation of fine toward coarse scales as local toward long‐range information integration, respectively. Beyond these temporal considerations, the spatial interpretation of the present findings remains constrained by the properties of scalp EEG and the low number of channels. Although the observed topography of effects was anatomically plausible, scalp potentials do not permit direct inference about precise cortical generators (Grech et al. 2008; Michel and Murray 2012). Relatedly, the use of a low‐density montage may have limited sensitivity to additional spatial features of learning‐related complexity changes beyond the midline frontal region. Particularly given the similar trend toward reduced complexity in frontocentral channels, the interpretation of findings should be seen as possible inferences based on previous findings and be done cautiously. Future studies would benefit from higher density EEG systems and source localization approaches to improve spatial specificity and to test whether comparable changes emerge in broader networks.

Finally, although MSE offers a promising framework for quantifying the temporal organization of neural activity, its physiological interpretation during movement execution still requires further empirical work. In relatively predictable and repetitively executed tasks, learning may be accompanied by a progressive reduction in large‐scale dynamics as control becomes increasingly economical. By contrast, tasks with higher unpredictability may place sustained demands on monitoring, potentially giving rise to distinct complexity profiles (Dayan and Cohen 2011; Guadagnoli and Lee 2004; Hardwick et al. 2013; Haith and Krakauer 2018; Piskin, Müller, et al. 2026). Future work should therefore compare complexity changes across motor tasks with different levels of predictability and feedback dependence to determine under which behavioral conditions reduced or preserved complexity reflects the most adaptive neural state.

## Author Contributions

**Daghan Piskin:** conceptualization, formal analysis, methodology, writing – original draft, visualization. **Linda Margraf:** conceptualization, data curation, methodology, writing – review and editing. **Matthias Weigelt:** conceptualization, writing – review and editing, supervision, project administration. **Jochen Baumeister:** writing – review and editing, supervision, project administration.

## Funding

The authors have nothing to report.

## Conflicts of Interest

The authors declare no conflicts of interest.

## Supporting information




**DATA S1:** Supporting Information.

## Data Availability

Data are available from the authors upon reasonable request.
